# Clinical and Pathological Characteristics and Prognosis of Breast Cancer During Pregnancy and Postpartum: A Multicenter Retrospective Study

**DOI:** 10.1002/cnr2.70577

**Published:** 2026-06-04

**Authors:** Liying Wang, Heran Deng, Xiaoyu Fang, TaoLang Li, Qianjun Chen, Jieqiong Liu

**Affiliations:** ^1^ Guangdong Provincial Key Laboratory of Malignant Tumor Epigenetics and Gene Regulation, Breast Tumor Center Sun Yat‐Sen Memorial Hospital, Sun Yat‐Sen University Guangzhou Guangdong China; ^2^ Department of Breast and Thyroid Surgery The Affiliated Hospital of Zunyi Medical University, Zunyi Medical University Zunyi Guizhou China; ^3^ Department of Breast Oncology Traditional Chinese Medicine Hospital of Guangdong Province, Guangzhou University of Chinese Medicine Guangzhou Guangdong China

**Keywords:** LASSO regression, postpartum breast cancer, pregnancy during breast cancer, retrospective study

## Abstract

**Purpose:**

The prognostic impact of the interval between the most recent childbirth and breast cancer diagnosis remains insufficiently defined, particularly when breast cancer diagnosed during pregnancy (PrBC) is distinguished from postpartum breast cancer (PPBC). In this multicenter Chinese cohort, we aimed to validate and extend prior observations regarding the association between time since recent childbirth, clinicopathological features, and outcomes.

**Methods:**

This multicenter retrospective cohort study included 12 453 patients aged 18–55 years with stage I to IV breast cancer for descriptive analyses; stage‐restricted sub‐cohorts were used for specific survival and modeling analyses as described below. These patients were diagnosed at three hospitals from July 1989 to June 2023, followed up until November 2023. The main exposures were diagnoses during pregnancy or the interval between the most recent childbirth and the diagnoses. The two primary outcomes assessed were disease‐free survival (DFS) and breast cancer‐specific death (BCSS). Cox proportional hazard models were employed to examine associations between exposures and outcomes. A nomogram using the least absolute shrinkage and selection operator (LASSO) penalty for variable selection. Model performance was evaluated using area under curve (AUC), decision curve analysis (DCA) curves and calibration.

**Results:**

Among 12 453 patients, 113 (0.9%) were diagnosed during pregnancy and 12 340 after childbirth. Patients diagnosed within 5 years after childbirth showed more aggressive clinicopathological characteristics, including larger tumors, more lymph node metastasis, and a higher Ki67 index. In contrast, results for PrBC should be interpreted cautiously because of the small sample size and potential treatment‐related confounding. The poor prognosis of PPBC is potentially linked to an increased risk of liver and brain metastases. Multivariate analysis indicates that a short interval between childbirth and diagnosis is an independent risk factor for breast cancer prognosis. Predictors included in the nomogram were time interval between childbirth and diagnosis of breast cancer, T stage, N stage, and pathological subtype. The AUCs for 1‐, 3‐, and 5‐year DFS prediction were 0.819, 0.767, and 0.731 in the training cohort and 0.818, 0.748, and 0.735 in the validation cohort.

**Conclusion:**

Patients diagnosed with PPBC within 10 years after their most recent pregnancy, particularly within 5 years, had increased risks of recurrence, metastasis, and mortality.

**Abbreviations**
AUC: areas under the curves
BCSS: breast cancer‐specific survival
CI: confidence interval
DCA: decision curve analysis
DFS: disease‐free survival
ER: estrogen receptor
HER2: human epidermal growth factor receptor 2
HR: hazard ratio
LASSO: the least absolute shrinkage and selection operator
PABC: pregnancy‐associated breast cancer
PD‐1: programmed cell death protein 1
PD‐L1: programmed cell death ligand 1
PPBC: postpartum breast cancer
PR: progestogen receptor
PrBC: breast cancer during pregnancy
tdROC: time‐dependent receiver operating characteristic curve

## 1 Background

Breast cancer is linked to the duration of exposure to female hormones [1, 2], with reproductive factors such as parity, age at first childbirth, breastfeeding, and genetic factors influencing the disease [3]. Pregnancy‐associated breast cancer (PABC) refers to breast cancer diagnosed during or within 1 year postpartum [4]. Increasing evidence supports separating breast cancer during pregnancy (PrBC) from postpartum breast cancer (PPBC) because of differences in biology and prognosis [5]. Recent data suggest that the adverse prognostic effect associated with postpartum breast cancer may extend beyond the first postpartum year and remain clinically relevant for up to 5–10 years after childbirth [6]. PrBC accounts for about 4% of breast cancers in women younger than 45 years [7]. The prognosis of PrBC remains a distinct clinical question and should not be conflated with postpartum breast cancer. Recent evidence suggests that when patients with PrBC receive stage‐appropriate, timely treatment, their outcomes appear comparable to those of non‐pregnant patients, whereas postpartum breast cancer shows a more consistent association with adverse metastatic and survival outcomes [8]. Lyons et al. indicated worse survival due to genetic differences [4], while a large cohort study found similar outcomes to breast cancer in non‐pregnant women [9].

In contrast, PPBC is increasingly recognized as a high‐risk subtype associated with inferior outcomes. Although historically defined within 1‐year postpartum, recent data suggest that the associated risk extends up to 5–10 years after childbirth. PPBC accounts for 35%–55% of breast cancer in women under 45 [10] and Amant et al. showed that PPBC was associated with poorer survival and higher metastasis risks than PrBC or in non‐pregnant women [11], independent of TNM stage and hormone receptor (HR) status [12]. Mechanistically, these adverse outcomes have been linked to postpartum mammary gland involution (PMI), a process characterized by tissue remodeling, inflammation, and immune suppression [13, 14, 15]. The involution microenvironment exhibits features of wound healing, including extracellular matrix remodeling [16, 17], lymph angiogenesis [18], and infiltration of immunosuppressive cells [19, 20], which may promote tumor dissemination and metastasis [21]. Together, these findings support PPBC as a biologically distinct entity rather than a temporal extension of PrBC.

Despite these advances, important gaps remain. Most evidence regarding PPBC risk windows and prognosis has come from Western populations, while data from Asian populations remain limited. In addition, prior studies have frequently combined PrBC and PPBC under a single PABC definition, potentially obscuring biologic and prognostic heterogeneity [22]. Clarifying this distinction is clinically relevant, because PrBC patients often require individualized management that takes gestational age and fetal safety into account, while PPBC treatment resembles non‐pregnant breast cancer [23]. And when diagnosed and treated per standard protocols, PrBC generally does not result in worse survival outcomes compared to age‐matched non‐pregnant patients, as shown by recent cohort analyses [24]. Ongoing research is exploring chemotherapy, radiotherapy for PrBC and immunotherapy for PPBC [9, 25]. Recognizing parity status as a prognostic factor for poor outcomes in premenopausal women with PPBC is crucial for optimizing treatment strategies [26]. At the same time, increasing evidence suggests that reproductive stage may also reflect underlying biological differences related to pregnancy, lactation, and involution, with potential implications for tumor biological signaling pathways [13, 27]. Emerging evidence supports the prognostic relevance of postpartum timing and related clinical factors. In a recent PPBC cohort, shorter postpartum intervals (< 2 years) and concurrent breastfeeding were associated with later stage at diagnosis and reduced survival compared with diagnoses made at later postpartum intervals, highlighting the importance of postpartum timing in outcome stratification [28]. Therefore, in this multicenter retrospective study of a Chinese population, we aimed to validate and refine prior observations regarding whether the interval from the most recent childbirth to breast cancer diagnosis is associated with adverse clinicopathologic features and outcomes, and whether separating PrBC from postpartum subgroups provides clinically meaningful prognostic stratification.

## 2 Methods

### 2.1 Participants and Exposure

This current study utilized databases from Sun Yat‐sen Memorial Hospital, Guangdong Traditional Chinese Medicine Hospital, and Affiliated Hospital of Zunyi Medical University. The dataset included women diagnosed with breast cancer between July 1, 1989, and June 30, 2023, aged 18–55 at diagnosis, with follow‐up until death or October 31, 2023. Exclusion criteria are detailed in Figure S1 in the Supplement. Patients were excluded if key exposure or outcome‐related variables were unavailable, including time from most recent childbirth to diagnosis, stage information, survival status, or follow‐up time. Because these variables were essential for the primary analyses, complete‐case analysis was used, and multiple imputation was not performed. Patients with inflammatory breast cancer were excluded to reduce potential heterogeneity related to the highly aggressive clinicopathological subtype and its distinct presentation, which may complicate analyses focused on the prognostic effect of childbirth‐related timing. Notably, recent data suggest that postpartum status did not significantly alter survival outcomes among IBC patients, likely because the inherently aggressive biology of IBC may overwhelm modest postpartum‐associated risks [29]. The main exposures were diagnoses during pregnancy or the interval between the most recent childbirth and the diagnoses. The present analysis was intentionally restricted to patients with documented pregnancy or childbirth‐related reproductive information relevant to the study question, because our primary objective was to evaluate prognostic heterogeneity according to timing since the most recent childbirth. Therefore, a nulliparous comparator group was not included in the current analytic dataset.

### 2.2 Outcomes

The primary outcomes included disease‐free survival (DFS) and breast cancer‐specific survival (BCSS). DFS was defined as the time from diagnosis to the first occurrence of locoregional recurrence, distant metastasis, or death from any cause, whichever occurred first. BCSS refers to the time from diagnosis to the date of death attributed to breast cancer. Individuals without disease recurrence or death from breast cancer were censored either at their last contact date or at the date of death from other causes, up to the study's cutoff date.

### 2.3 Statistical Analyses

Patients were categorized into four groups according to reproductive timing at diagnosis: breast cancer diagnosed during pregnancy (PrBC), postpartum breast cancer diagnosed within 5 years after childbirth (PPBC ≤ 5 years), postpartum breast cancer diagnosed 5 to ≤ 10 years after childbirth (PPBC 5 to ≤ 10 years), and postpartum breast cancer diagnosed > 10 years after childbirth (PPBC > 10 years). These categories were prespecified to align with prior studies suggesting that the adverse prognostic effect of postpartum timing is most pronounced within the first 5 years and may remain clinically relevant up to 10 years after childbirth. The four groups were compared using the Kruskal‐Wallis test for continuous variables and Pearson's Chi‐square test for categorical variables. Descriptive analysis of distant metastatic sites compared their frequency distribution across different time intervals since the most recent childbirth. The Kaplan–Meier method was used to calculate DFS and BCSS, with survival differences assessed using the log‐rank test. Cox proportional hazard models identified prognostic factors for DFS and BCSS, estimating hazard ratios (HRs) with 95% confidence intervals (CIs). These models were adjusted for factors including age at diagnosis, TNM stage, pathological subtype (characterized by ER, PR, and HER2 status), and year of diagnosis. In subgroup analyses stratified by stage or ER status, the stratification variable was not re‐entered as a covariate.

Patients were randomly divided into training and validation sets in a 7:3 ratio. Candidate predictors were first screened using LASSO Cox regression in the training cohort. Variables with non‐zero coefficients were then entered into a multivariable Cox model, and predictors that remained independently associated with DFS were used to construct the nomogram. Time‐dependent receiver operating characteristic (tdROC) curves were plotted, with areas under curves (AUCs) at 1, 3, and 5 years calculated to assess model discrimination. Internal validation was performed using the Bootstrap method. Calibration curves assessed survival prediction accuracy, and decision curve analysis (DCA) evaluated the clinical utility. Data were analyzed using STATA MP and the R (version 4.2.2). The *p*‐values less than 0.05 in two‐sided tests were deemed statistically significant.

## 3 Results

### 3.1 Clinicopathological Characteristics

Between July 1, 1989, and June 30, 2023, the records of 17 700 patients were retrospectively screened. Among these, 12 453 patients met the eligibility criteria and were included in the final analyses (Figure S1 in the Supplement). The clinical and pathological characteristics of these patients were summarized in Table 1 categorized by the interval between the last childbirth and the breast cancer diagnosis.

**TABLE 1:** Clinicopathological characteristics of breast cancer patients during pregnancy and postpartum.

Characteristic	No. (%) (*N* = 12 453)				*p* value^a^
	PrBC	PPBC ≤ 5 years	PPBC 5 to ≤ 10 years	PPBC > 10 years	
No. (%)	*N* = 113 (0.9)	*N* = 1005 (8.1)	*N* = 1824 (14.6)	*N* = 9511 (76.4)	
Age group					< 0.001
≤ 40	102 (90.3)	912 (90.7)	1533 (84.0)	756 (8.0)	
40–55	11 (9.7)	93 (9.3)	291 (16.0)	8746 (92.0)	
Mean (SD)	33.8 (4.7)	33.5 (4.7)	37.4 (3.69)	47.2 (4.6)	
Median (range)	33 (23–45)	32 (21–49)	37 (26–53)	47 (33–55)	
Age at menarche					< 0.001
< 13	25 (22.1)	206 (20.5)	335 (18.4)	1219 (12.8)	
≥ 13	88 (77.9)	799 (79.5)	1489 (81.6)	8292 (87.2)	
Parity					< 0.001
1	54 (47.8)	512 (50.9)	1015 (55.6)	6629 (69.7)	
2	46 (40.7)	389 (38.7)	653 (35.8)	2048 (21.5)	
3 or more	13 (11.5)	104 (10.3)	156 (8.6)	834 (8.8)	
Last delivery age					< 0.001
Mean (SD)	33.6 (4.8)	30.1 (4.5)	28.6 (3.4)	26.2 (1.9)	
< 30	26 (23.0)	579 (57.6)	1308 (71.7)	9022 (94.9)	
≥ 30	87 (77.0)	426 (42.4)	516 (28.3)	489 (5.1)	
T stage					< 0.001
T0	7 (6.2)	75 (7.5)	177 (9.7)	860 (9.0)	
T1	34 (30.1)	409 (40.7)	862 (47.3)	4551 (47.8)	
T2	45 (39.8)	409 (40.7)	662 (36.3)	3462 (36.4)	
T3	19 (16.8)	74 (7.4)	64 (3.5)	346 (3.6)	
T4	4 (3.5)	20 (2.0)	31 (1.7)	172 (1.8)	
Unknown	4 (3.5)	18 (1.8)	28 (1.5)	120 (1.3)	
N stage					< 0.001
N0	56 (49.6)	574 (54.4)	1102 (60.4)	5863 (61.5)	
N1	27 (23.9)	273 (27.2)	430 (23.6)	2245 (23.6)	
N2	14 (12.4)	105 (10.4)	175 (9.6)	813 (8.5)	
N3	15 (13.3)	73 (7.3)	105 (5.8)	528 (5.6)	
Unknown	1 (0.9)	7 (0.7)	12 (0.7)	72 (0.8)	
M stage					< 0.001
M0	103 (91.2)	958 (95.3)	1787 (98.0)	9283 (97.6)	
M1	10 (8.8)	47 (4.7)	37 (2.0)	228 (2.4)	
Clinical stage					< 0.001
Tis	7 (6.2)	72 (7.2)	166 (9.1)	796 (8.4)	
I	21 (18.6)	263 (26.2)	591 (32.4)	3257 (34.2)	
II	46 (40.7)	435 (43.3)	725 (39.7)	3768 (39.6)	
III	26 (23.0)	171 (17.0)	278 (15.2)	1343 (14.1)	
IV	10 (8.8)	47 (4.7)	37 (2.0)	228 (2.4)	
Unknown	3 (9.1)	17 (1.7)	27 (1.5)	119 (1.3)	
Histologic subtype					0.029
Ductal	98 (86.7)	935 (93.0)	1674 (91.8)	8745 (91.9)	
Lobular	3 (2.7)	14 (1.4)	46 (2.5)	285 (3.0)	
Inflammatory	0 (0.0)	1 (0.1)	0 (0.0)	2 (0.0)	
Other	12 (10.6)	55 (5.5)	104 (5.7)	479 (5.0)	
ER status					0.512
Negative	25 (22.1)	201 (20.0)	331 (18.1)	1812 (19.1)	
Positive	86 (76.1)	797 (79.3)	1478 (81.0)	7626 (80.2)	
Unknown	2 (1.8)	7 (0.7)	15 (0.8)	73 (0.8)	
PR status					0.001
Negative	46 (40.7)	315 (31.3)	484 (26.5)	2794 (29.4)	
Positive	65 (57.5)	677 (74.2)	1314 (72.0)	6620 (69.6)	
Unknown	2 (1.8)	13 (1.3)	26 (1.4)	97 (1.0)	
HER2 status					0.256
Negative	64 (56.6)	661 (65.8)	1188 (65.1)	6208 (65.3)	
Positive	41 (36.3)	294 (29.3)	516 (28.3)	2675 (28.1)	
Unknown	8 (7.1)	50 (5.0)	120 (6.6)	628 (6.6)	
Ki67 expression					0.001
< 20%	31 (27.4)	277 (27.6)	595 (32.6)	3169 (33.3)	
≥ 20%	67 (59.3)	636 (63.3)	1044 (57.2)	5436 (57.2)	
Unknown	15 (13.3)	92 (9.1)	185 (10.2)	906 (9.5)	
Biologic subtype					< 0.001
Luminal A^b^	17 (15.0)	167 (16.6)	399 (21.9)	2125 (22.3)	
Luminal B^c^	69 (61.1)	630 (62.7)	1079 (59.2)	5501 (57.8)	
HER2+^d^	8 (13.1)	69 (6.9)	130 (7.1)	748 (7.9)	
Triple negative^e^	13 (11.5)	102 (10.1)	134 (7.3)	729 (7.7)	
Unknown	6 (12.3)	37 (3.7)	82 (4.5)	408 (4.3)	
Chemotherapy					< 0.001
Yes	97 (85.8)	880 (87.6)	1447 (79.3)	7273 (76.5)	
No	13 (11.5)	108 (10.7)	312 (17.1)	1794 (18.9)	
Unknown	3 (2.7)	17 (1.7)	65 (3.6)	444 (4.7)	
Radiotherapy					< 0.001
Yes	72 (63.7)	695 (69.2)	1244 (68.2)	6188 (65.1)	
No	34 (30.1)	278 (27.7)	535 (29.3)	3126 (23.2)	
Unknown	7 (6.2)	32 (3.2)	45 (2.5)	197 (2.1)	
Endocrine therapy					0.063
Yes	73 (64.6)	682 (67.9)	1300 (71.3)	6759 (71.1)	
No	40 (35.4)	318 (31.6)	519 (28.5)	2693 (28.3)	
Unknown	0 (0.0)	5 (0.5)	5 (0.3)	59 (0.6)	
Surgery type					0.146
Breast conserving Surgery	46 (40.7)	480 (47.8)	901 (49.4)	4477 (47.1)	
Unilateral mastectomy	57 (50.4)	481 (47.9)	874 (47.9)	5774 (50.2)	
Bilateral mastectomy	4 (3.5)	25 (2.5)	28 (1.5)	186 (2.0)	
Unknown	6 (5.3)	19 (1.9)	21 (1.2)	74 (0.8)	
BC family history					0.023
Yes	7 (6.2)	58 (5.8)	103 (5.6)	413 (4.3)	
No	105 (92.9)	935 (93.0)	1701 (93.3)	8996 (94.6)	
Unknown	1 (0.9)	12 (1.2)	20 (1.1)	102 (1.1)	
Year of diagnosis					0.006
1989–1998	1 (0.9)	2 (0.2)	2 (0.1)	7 (0.1)	
1999–2004	2 (1.8)	32 (3.2)	61 (3.3)	288 (3.0)	
2005–2014	14 (12.4)	157 (15.6)	346 (19.0)	1872 (19.7)	
2015–2023	96 (85.0)	814 (81.0)	1415 (77.6)	7344 (77.2)	

Abbreviations: −, negative; +, positive; ER, estorgen receptor; HER2, human epidermal growth factor receptor 2; PPBC, postpartum breast cancer; PR, progesterone receptor; PrBC, breast cancer during pregnancy.

^a^ Kruskal‐Wallis test for continuous variables and Pearson's Chi‐square test for categorical variables. Missing categories were excluded from statistical analysis.

^b^ Luminal A defined as ER+, PR+, HER2−, Ki67 < 20%.

^c^ Luminal B defined as ER+, PR−/+, HER2−/+, Ki67 any.

^d^ HER2+ defined as ER−, PR−, and HER2+.

^e^ Triple negative defined as ER−, PR−, and HER2−.

Patients diagnosed with PPBC within 5 years showed the most consistent pattern of adverse clinicopathological features. Findings in the PrBC subgroup should be interpreted cautiously because of the small sample size and possible treatment‐related confounding. They are younger, have earlier menarche, and have had two or more childbirths. Both groups show higher rates of ER‐negative tumors (22.1% and 20.0%), larger tumor sizes (> 5 cm in 20.3% and 9.4%), more lymph node metastasis (49.6% and 44.9%), and higher TNM stages (31.8% and 21.7% at stages III and IV). The prevalence of TNBC was also higher in these groups (11.5% and 10.1%), with a notably higher Ki67 index in patients within 5 years postpartum (63.3%). These patients were more likely to receive chemotherapy (87.6%), radiotherapy (69.2%), and preferred breast‐conserving surgery (47.8%). They also had a higher proportion of family history of breast cancer (*p* = 0.023). Of the 12 453 patients, 551 were lost to follow‐up, resulting in a retention rate of 95%.

### 3.2 Analysis of Risk Factors Associated With Distant Metastasis in PPBC

In a cohort study of 10 922 patients with stage I to III breast cancer who were evaluable for analyses of metastatic risk‐related clinicopathological characteristics, we assessed tumor size, lymph node involvement, and Ki67 index according to time from most recent childbirth to diagnosis. The study found that PrBC patients and those diagnosed with PPBC within five years exhibited the largest median tumor diameter, 3.51 cm and 2.83 cm, respectively (Figure S2A in the Supplement). This difference was particularly pronounced in stage II patients. Among stage III tumors, only those diagnosed with PPBC within 5 years had a significantly larger median tumor diameter compared to those diagnosed with PPBC > 10 years (*p* < 0.05, Figure S2D in the Supplement). Additionally, patients diagnosed with PPBC within five years showed a higher rate of lymph node metastasis than those diagnosed with PPBC > 10 years (*p* < 0.001). However, no significant difference in lymph node positivity rates was observed between PrBC patients and those diagnosed with PPBC five to ten years post‐delivery, with rates of 50.0% and 42.3% (*p* = 0.07 and *p* = 0.26; Figure S2B in the Supplement). We also observed a decrease in the Ki67 index with increasing time since delivery. Patients with PrBC and those diagnosed with PPBC within five years exhibited higher average Ki67 values compared to those diagnosed > 10 years (*p* < 0.0001, Figure S2C in the Supplement).

### 3.3 Enrichment of Distant Metastasis Characteristics Patterns

**FIGURE 1:**
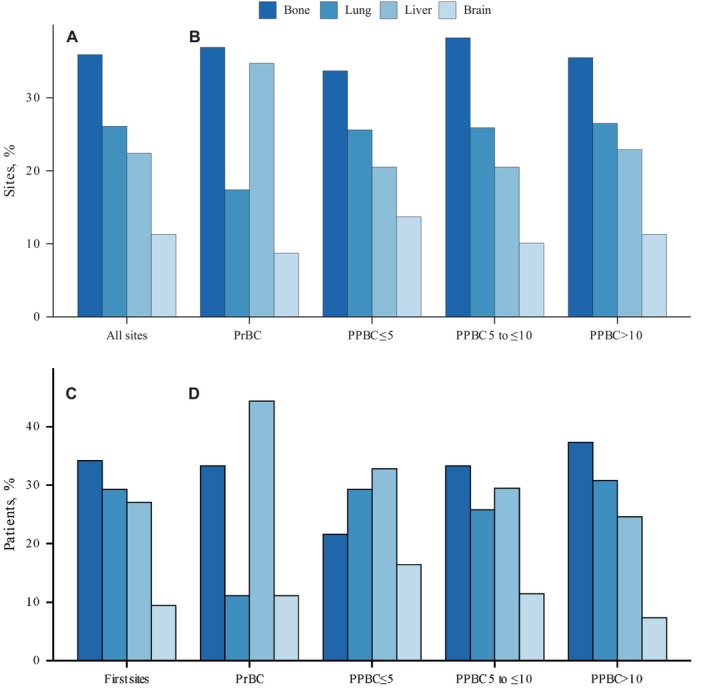
Distribution of Distant Metastasis Sites according to time from most recent childbirth to breast cancer diagnosis. (A) Total distribution of all recorded metastatic lesions among patients with distant metastases and known metastatic sites (*N* = 797). (B) Distribution of metastatic lesions stratified by reproductive group: PrBC, PPBC ≤ 5 years, PPBC 5 to ≤ 10 years, and PPBC > 10 years. (C) Distribution of first distant metastatic sites among patients with a documented initial metastatic event. (*N* = 786). (D) Distribution of first distant metastatic sites stratified by reproductive group: PrBC, PPBC ≤ 5 years, PPBC 5 to ≤ 10 years, and PPBC > 10 years. PrBC, breast cancer diagnosed during pregnancy; PPBC, postpartum breast cancer. The number of evaluable patients for each analysis is indicated in the figure or legend.

Among 845 patients who developed distant metastasis, 797 had at least one documented metastatic site and were included in the analysis of overall metastatic site distribution. Of these, 786 also had a clearly documented first distant metastatic site and were included in the analysis of initial metastatic pattern. We found the bone was the most common site for both metastatic and initial metastasis (614 lesions [35.9%]; 269 patients [34.2%]; Figure 1 and Table S1 in the Supplement).

Descriptively, liver metastasis appeared more frequent in the PrBC group (16 lesions [34.7%]), and brain/nervous system metastasis (30 lesions [13.7%]) appeared more frequent in patients diagnosed within 5 years postpartum. (Figure 1 and Table S1 in the Supplement). However, these subgroup analyses were based on limited event numbers and should be interpreted as hypothesis‐generating rather than confirmatory.

Analysis revealed that PrBC patients and those diagnosed with PPBC within 5 years exhibited a higher tendency for initial liver metastasis compared to PPBC > 10 years (*p* = 0.092 and *p* = 0.071), consistent with previous studies [30, 31]. Moreover, patients diagnosed with PPBC within 5 years were more likely to experience initial brain and nervous system metastasis, while patients diagnosed > 10 years were less likely to have initial bone metastasis (*p* = 0.001; Figure 1 and Table S1 in the Supplement). In the TNBC cohort, PrBC patients were more likely to experience initial liver metastasis compared to PPBC > 10 years (*p* = 0.046), while PPBC patients had a higher propensity for brain metastasis within the first 5 years (*p* = 0.048; Table S2 in the Supplement).

### 3.4 Association of Childbirth and Time Since Recent Childbirth With Outcomes

Among 11 963 patients, 10.3% (1235 individuals) experienced recurrence and distant metastases (Figure S3 in the Supplement).

Patients diagnosed with PPBC within 5 years had a significantly higher risk of DFS events (HR = 2.15, 95% CI 1.92–2.55, *p* < 0.001), and breast cancer‐specific mortality (HR = 1.70, 95% CI 1.32–2.19, *p* < 0.001) compared to those diagnosed more than 10 years postpartum. This increased risk persisted even after adjusting for covariates (adjusted HR = 2.69, 95% CI 2.09–3.47, *p* < 0.001; adjusted HR = 2.12, 95% CI 1.46–3.08, *p* < 0.001). This poor prognosis continued for up to 10 years (HR = 1.41, 95% CI 1.21–1.64, *p* < 0.001; adjusted HR = 1.65, 95% CI 1.35–2.03, *p* < 0.001), suggesting that diagnosis within 10 years after recent childbirth, especially within the first 5 years, is associated with poorer prognosis. In contrast, PrBC patients showed elevated recurrence and metastasis risks (HR = 2.92, 95% CI 1.90–4.51, *p* < 0.001), but no increase in BCSS (HR = 1.59, 95% CI 0.71–3.57, *p* = 0.257) (Table 2 and Figure S4A,B in the Supplement).

**TABLE 2:** Univariate and multivariate cox proportional hazard regression for DFS and BCSS.

PPBC time, year	Disease‐free survival				Breast cancer‐specific survival			
	Univariate		Multivariate		Univariate		Multivariate	
	HR (95% CI)	*p* value	HR (95% CI)	*p* value	HR (95% CI)	*p* value	HR (95% CI)	*p* value
All stages^a^								
> 10	1 [Reference]	NA	1 [Reference]	NA	1 [Reference]	NA	1 [Reference]	NA
5 to ≤ 10	1.41 (1.21–1.64)	**< 0.001**	1.65 (1.35–2.03)	**< 0.001**	1.26 (1.01–1.56)	**0.037**	1.48 (1.20–2.00)	**0.010**
≤ 5	2.15 (1.82–2.55)	**< 0.001**	2.69 (2.09–3.47)	**< 0.001**	1.70 (1.32–2.19)	**< 0.001**	2.12 (1.46–3.08)	**< 0.001**
PrBC	2.92 (1.90–4.51)	**< 0.001**	3.33 (2.07–5.35)	**< 0.001**	1.59 (0.71–3.57)	0.257	1.79 (0.76–4.21)	0.181
Stage 0–II^b^								
> 10	1 [Reference]	NA	1 [Reference]	NA	1 [Reference]	NA	1 [Reference]	NA
5 to ≤ 10	1.42 (1.18–1.71)	**< 0.001**	1.67 (1.30–2.14)	**< 0.001**	1.28 (0.97–1.70)	0.085	1.72 (1.17–2.53)	**0.006**
≤ 5	2.36 (1.92–2.91)	**< 0.001**	2.97 (2.18–4.03)	**< 0.001**	1.55 (1.09–2.21)	**0.015**	2.44 (1.49–4.00)	**< 0.001**
PrBC	2.81 (1.54–5.10)	**< 0.001**	3.76 (1.99–7.13)	**< 0.001**	0.57 (0.08–4.05)	0.573	0.95 (0.13–7.01)	0.962
Stage III^b^								
> 10	1 [Reference]	NA	1 [Reference]	NA	1 [Reference]	NA	1 [Reference]	NA
5 to ≤ 10	1.41 (1.10–1.82)	**0.008**	1.53 (1.07–2.19)	**0.020**	1.24 (0.88–1.73)	0.216	1.22 (0.76–1.96)	0.413
≤ 5	1.66 (1.23–2.24)	**< 0.001**	2.37 (1.51–3.71)	**< 0.001**	1.69 (1.17–2.44)	**0.005**	1.91 (1.07–3.40)	**0.029**
PrBC	2.07 (1.10–3.90)	**0.023**	2.93 (1.44–5.94)	**0.003**	1.56 (0.64–3.79)	0.328	2.03 (0.75–5.50)	0.162
ER+^c^								
> 10	1 [Reference]	NA	1 [Reference]	NA	1 [Reference]	NA	1 [Reference]	NA
5 to ≤ 10	1.46 (1.23–1.73)	**< 0.001**	1.81 (1.44–2.27)	**< 0.001**	1.28 (1.00–1.65)	0.050	1.58 (1.14–2.20)	0.070
≤ 5	2.22 (1.83–2.70)	**< 0.001**	2.80 (2.10–3.72)	**< 0.001**	1.92 (1.44–2.55)	**< 0.001**	2.36 (1.56–3.57)	**< 0.001**
PrBC	2.98 (1.79–4.98)	**< 0.001**	3.48 (1.99–6.09)	**< 0.001**	1.56 (0.58–4.19)	0.375	1.68 (0.59–4.76)	0.328
ER−^c^								
> 10	1 [Reference]	NA	1 [Reference]	NA	1 [Reference]	NA	1 [Reference]	NA
5 to ≤ 10	1.26 (0.92–1.73)	**0.145**	1.30 (0.87–1.95)	0.203	1.21 (0.79–1.85)	0.385	1.12 (0.64–1.95)	0.688
≤ 5	1.91 (1.35–2.69)	**< 0.001**	2.26 (1.37–3.72)	**0.001**	1.11 (0.62–1.96)	0.732	1.15 (0.54–2.47)	0.714
PrBC	2.80 (1.24–6.30)	**0.013**	3.04 (1.27–7.31)	**0.013**	1.63 (0.40–6.59)	0.494	1.62 (0.37–7.11)	0.521

*Note:* The bold values indicate statistical significance with *p* < 0.05.

^a^ Adjusted for age at diagnosis, TNM stage, type and year at diagnosis.

^b^ Adjusted for age at diagnosis, type and year at diagnosis.

^c^ Adjusted for age at diagnosis, TNM stage, and year at diagnosis.

**FIGURE 2:**
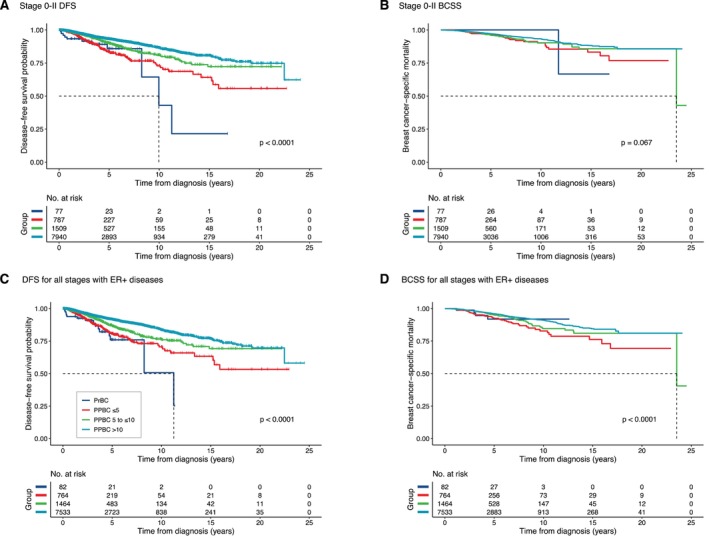
Disease‐free survival and breast cancer‐specific survival according to time from most recent childbirth to diagnosis in selected subgroups. (A) Disease‐free survival in patients with stage 0–II disease. (B) Breast cancer‐specific survival in patients with stage 0–II disease. (C) Disease‐free survival in patients with ER‐positive tumors. (D) Breast cancer‐specific survival in patients with ER‐positive tumors. Hazard ratios were estimated using Cox proportional hazards models with covariate adjustment as specified in Table 2.

Furthermore, our findings indicate that patients diagnosed with PPBC within 5 years, particularly those in stages I and II or ER‐positive tumors, exhibited a 1.5 to 2 times higher risk of recurrence, distant metastasis, and mortality (Figure 2A–D and Table 2). Conversely, no significant statistical differences were observed in patients with stage III or ER‐negative tumors, possibly attributed to the smaller sample size (Table 2 and Figure S4C–F in the Supplement).

### 3.5 Establishment of Prognostic Nomogram

**FIGURE 3:**
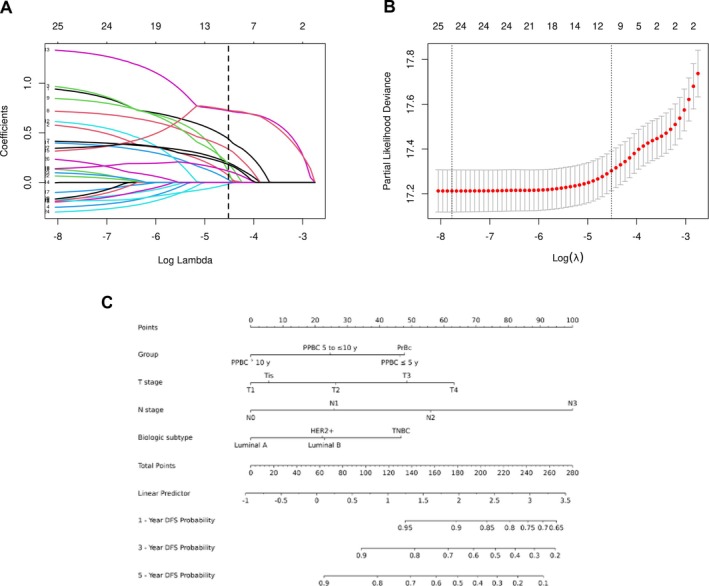
Establishment of the disease‐free survival‐nomogram. (A) LASSO coefficient profiles of the clinical features. (B) Optimal feature selection of cross‐validation. (C) Nomogram to predict the 1, 3 and 5‐year DFS. The nomogram was developed in the training cohort and evaluated in the validation cohort.

A total of 11 963 stage 0 to III breast cancer patients were randomly divided into the training set (*n* = 8374) and the validation set (*n* = 3589) in a 7:3 ratio. There were no statistically significant differences in baseline clinicopathological characteristics between the two groups (Table S3 in the Supplement). Pregnancy and postpartum group, T stage, N stage, clinical stage, PR status, Ki67 expression, and pathological subtypes were selected to construct the model after LASSO cox regression and tenfold cross‐validation (Figure 3). The details of these variables are shown in Figure S5 in the Supplement. Multivariate cox proportional hazard regression analysis identified 4 of 7 factors (*p* < 0.05) as significant predictors of DFS (Table S4 in the Supplement). The DFS nomogram is shown in Figure 3. The aim of model construction was to explore whether timing since recent childbirth may add prognostic information beyond standard clinicopathological factors; however, formal evaluation of incremental predictive gain requires further external validation.

### 3.6 Assessment and Clinical Application of Prognostic Nomogram

The AUCs of 1‐year, 3‐year, and 5‐year DFS for patients in the training set were 81.9% (95% CI 78.3–85.4), 76.7% (95% CI 74.2–79.2), and 73.1% (95% CI 70.8–75.4). In the validation set, the AUCs for 1‐year, 3‐year, and 5‐year DFS were 81.8% (95% CI 75.5–88.2), 74.8% (95% CI 71.0–78.7), and 73.5% (95% CI 69.9–77.0). All AUCs were greater than 0.7, indicating good model discrimination and moderate prediction accuracy (Figure S6 in the Supplement). The calibration curves for predicting the 1‐year, 3‐year, and 5‐year survival rates of breast cancer patients closely aligned with the actual probabilities, indicating good model consistency (Figure S7 in the Supplement). Clinical decision curve analysis (DCA) showed threshold probabilities for DFS at 1‐year, 3‐year, and 5‐year intervals, ranging from 3% to 38%, 4% to 55%, and 10% to 74%. Consequently, the strategy of screening high‐risk populations using our nomogram was higher than that of no screening or full screening strategies (Figure S8 in the Supplement).

## 4 Discussion

This multicenter retrospective study focused on analyzing the clinical pathological differences and prognosis between PrBC and PPBC. Our findings add evidence from a large Asian multicenter cohort and broadly support prior studies suggesting that early postpartum timing is associated with more aggressive disease behavior. We observed that patients diagnosed within 5 years postpartum exhibit more aggressive pathological features and a higher risk of distant metastasis, especially to the liver or brain, compared to those diagnosed with breast cancer more than 10 years postpartum, whereas findings in the PrBC subgroup were less robust and should be interpreted cautiously. After adjusting for age, TNM stage, pathological subtypes and year of diagnosis, patients diagnosed within 10 years postpartum had nearly double the risk of recurrence or metastasis. A short interval between recent childbirth and diagnosis is significantly associated with higher rates of recurrence, distant metastasis, and mortality, even in stage I to II or ER‐positive tumors. These differences in tumor subtype distribution should be interpreted cautiously, as the younger age structure of the PrBC and early PPBC groups may also contribute to the higher proportion of ER‐negative disease. Based on multivariate analyses, we developed a nomogram with good predictive accuracy and reliability in evaluating the risk of breast cancer recurrence and metastasis. Therefore, the interval following recent childbirth should be considered a crucial prognostic factor in clinical practice.

Studies on rodent models and the postpartum breast microenvironment in women have shown wound healing‐like alterations during postpartum mammary gland involution (PMI) [20, 30, 32, 33, 34, 35]. This involves lymph‐angiogenesis, vascular infiltration, and lymph node involvement, which may increase the risk of distant metastasis [16, 36, 37]. Our findings support the theory that lymph node involvement and Ki67 expression decrease over time postpartum [38, 39]. We observed a high incidence of lymph node involvement in breast cancer patients within 10 years postpartum, particularly within 5 years [18], suggesting that breast lymphatic vessels expansion may persist for up to a decade after pregnancy. This highlights the role of interactions between tumor cells and postpartum lymphatic vessels in metastasis efficiency [38, 40].

Beyond tissue remodeling and lymphangiogenesis, recent molecular studies suggest that postpartum breast cancer may also have distinct biological features, including enhanced cell‐cycle activity, altered immune‐related signaling and cellular infiltration, and reduced estrogen receptor‐related signaling. These findings support the interpretation that postpartum timing may capture biologically meaningful heterogeneity not fully reflected by conventional clinicopathological variables alone [41].

Our descriptive findings suggest a possible enrichment of liver metastasis in PrBC and early PPBC, but these observations did not consistently reach statistical significance and should therefore be interpreted cautiously as hypothesis‐generating. The liver was identified as the primary site for initial metastasis in patients with PrBC and those diagnosed within five years postpartum, consistent with previous studies [42, 43, 44]. For example, Goddard et al. reported a 3.6‐fold increase in liver metastasis risk within five years postpartum [30]. Recent studies showed that the liver enlarges and enhances its synthetic metabolic output during pregnancy, then shrinks post‐weaning due to increased catabolic metabolism [17, 31]. These findings underscore the need for further investigation into how post‐weaning mammary involution may trigger liver metastasis and potential therapeutic targets [45, 46]. Furthermore, we observed a higher proportion of initial brain metastases in patients diagnosed within five years postpartum. This suggests that the worsening prognosis in these patients may be associated with an elevated risk of brain and nervous system metastases, a topic inadequately addressed in the literature [47]. Further exploration of the association between brain metastasis and the prognosis of PPBC [48].

Recent studies have indicated that PPBC has a significantly worse prognosis, while the diagnosis and treatment of PrBC do not increase the risk of disease progression and death [6, 49, 50]. Our results showed an increased hazard of DFS events in PrBC, whereas breast cancer‐specific mortality was not significantly increased. Therefore, the prognostic interpretation of PrBC in this cohort should remain cautious. Interpretation of PrBC outcomes is additionally limited by incomplete treatment‐timing information and the possibility of delayed systemic therapy during pregnancy, which may confound comparisons with postpartum groups in retrospective analyses. Previous studies have often combined the investigation of breast cancer diagnosed during pregnancy (PrBC) with that diagnosed within one year postpartum into a single study [51, 52]. Our results similarly demonstrated that while PrBC patients have a higher risk of recurrence and metastasis, no significant association with mortality was observed. In agreement with previous studies [53], we also indicated that patients diagnosed within five years postpartum face increased risks of recurrence and metastasis. Our retrospective data analysis showed that the average gestational age at diagnosis was 24 weeks, but only three patients received chemotherapy during pregnancy, suggesting that delays in treatment may complicate outcomes [49, 53]. A study highlighted that breast cancer patients face the highest risk of mortality within the first year postpartum (HR = 1.59, 95% CI 1.30–1.82), which may persist up to six years (HR = 1.14, 95% CI 0.99–1.25) [54]. Moreover, a meta‐analysis of 41 studies reported a higher mortality risk in PPBC patients (HR = 1.79, 95% CI 1.39–2.29) [12]. Some studies also suggest an independent association between ER‐positive tumors and increased mortality in PPBC patients [6, 49]. However, we find no associations between parity, age at menarche, or age at menopause and adverse outcomes. However, prior genomic sequencing literature revealed that older primiparous women have a higher probability of accumulating carcinogenic events in the epithelium compared to younger or nulliparous women [55]. Future studies should explore cancer risks across different reproductive age groups to better understand these disparities [56]. We also acknowledge that the exclusion of inflammatory breast cancer may be debated considering recent literature. Because the present study design and case definition were established before some of these newer reports became available, this decision should be interpreted as a methodological choice to reduce heterogeneity rather than as evidence that inflammatory breast cancer is unrelated to postpartum‐associated risk.

In this study, a LASSO cox regression model [57] was employed to develop a nomogram capable of calculating survival probabilities for each patient according to their predicted total score. This nomogram should be considered an exploratory prognostic model derived from retrospective data. A more granular categorization of the postpartum interval, particularly within the first year after childbirth, may provide additional clinical insight. However, in the present cohort, such further subdivision would have reduced the number of outcome events in each subgroup and may have led to unstable risk estimates, especially in stratified and model‐based analyses. Future studies with larger datasets should evaluate whether a separate PPBC < 1 year subgroup and exclusion of PrBC from prediction models improve prognostic precision. It may help illustrate the potential contribution of postpartum timing to recurrence risk stratification, but external validation and further methodological evaluation are required before clinical application.

This study has several limitations. The first one is the potential for selection bias in a retrospective study design. However, the study has a large sample size with low follow‐up loss and detailed survival data. Another limitation is the lack of several important reproductive variables, including breastfeeding duration and weaning time, which were unavailable or incomplete in the retrospective database and could not be incorporated into the present analyses. This limited our ability to directly link the observed prognostic patterns to postpartum involution‐related biological processes. While the study benefits from a substantial sample size and internal data validation, it is essential to conduct further prospective research or high‐quality randomized controlled trials to validate these results. In addition, the absence of a nulliparous comparison group limits direct comparison with prior population‐based studies and restricts causal interpretation of the prognostic effect of recent childbirth. Accordingly, our findings should be interpreted primarily as comparisons among parous women across different intervals since the most recent childbirth, rather than as estimates of risk relative to nulliparous women. Although a nulliparous comparator was not included, this design choice should be interpreted in the context of population‐based evidence from China showing that lifetime childlessness was 5.16% among women aged 49 years in 2020 [58, 59], indicating that most women at completed reproductive age are parous. Nevertheless, the absence of a nulliparous comparison group remains an important limitation because it restricts direct comparison with prior literature and limits causal interpretation of the prognostic effect of recent childbirth. The small number of PrBC cases is another important limitation, particularly for model development. Therefore, the contribution of PrBC to the exploratory nomogram should be interpreted cautiously. A more granular categorization of the postpartum interval, particularly within the first year after childbirth, may provide additional clinical insight and should be evaluated in future studies with sufficient event numbers. Because the cohort spans more than three decades, changes in imaging, pathology, and systemic treatment over time may have introduced residual era effects despite adjustment for year of diagnosis.

## 5 Conclusions

This multicenter study demonstrated that patients diagnosed with PPBC within 10 years after their most recent pregnancy had elevated risks of recurrence, metastasis, and mortality, regardless of ER status. These findings further confirm that PPBC may represent a distinct subgroup, emphasizing the necessity for distinct clinical and translational research approaches. It is crucial to conduct further research to uncover the underlying mechanisms between recent childbirth and adverse outcomes in breast cancer patients. By incorporating the timing of recent childbirth into clinical practice, the accuracy of prognosis for young breast cancer patients can be enhanced.

## Author Contributions

**Liying Wang:** conceptualization, methodology, software, data curation, formal analysis, investigation, validation, writing – original draft. **Heran Deng:** conceptualization, methodology, software, data curation, investigation, validation, formal analysis, writing – original draft. **Jieqiong Liu:** conceptualization, supervision, resources, project administration, visualization, funding acquisition, writing – original draft, writing – review and editing. **TaoLang Li:** conceptualization, writing – original draft, writing – review and editing, visualization, project administration, data curation. **Xiaoyu Fang:** writing – original draft, writing – review and editing, conceptualization, methodology, software, data curation, investigation, validation, formal analysis. **Qianjun Chen:** conceptualization, supervision, resources, project administration, visualization, funding acquisition, writing – original draft, writing – review and editing, data curation.

## Funding

This study was funded by the National Natural Science Foundation of China (82072906, 82160505), by the grant from Guangzhou Science and Technology Plan Project (2022A1515012238).

## Disclosure

This study adhered to the Strengthening the Reporting of Observational Studies in Epidemiology (STROBE) guidelines [60]. The database provides comprehensive information on newly diagnosed breast cancer cases and childbirth‐related attributes. Disease diagnoses were coded according to the Tenth Revision of the International Classification of Diseases, Clinical Modification (ICD‐10‐CM). Patient information was meticulously organized and securely stored using the Research Electronic Data Capture (REDCap) system. Follow‐up data were collected through a review of medical records and cross‐referencing with Hospital Registry data to validate the dates of metastasis, mortality, and the last recorded contact.

## Ethics Statement

This study obtained ethical approval from the Health Research Ethics Boards of the Sun Yat‐sen Memorial Hospital, the Traditional Chinese Medicine Hospital of Guangdong Province, and the Affiliated Hospital of Zunyi Medical University (approval numbers: SYSKY‐2023‐385‐01). Informed consent was waived for this retrospective study.

## Consent

The authors have nothing to report.

## Conflicts of Interest

The authors declare no conflicts of interest.

## Supporting information


**Figure S1:** Study profile.
**Figure S2:** Analysis of known clinical risk factors associated with distant metastasis in PPBC.
**Figure S3:** Association of childbirth and time since recent childbirth with outcomes.
**Figure S4:** Association of childbirth and time since recent childbirth with outcomes by stage or ER status.
**Figure S5:** Histogram of the coefficients of the selected features.
**Figure S6:** AUCs of the prediction models.
**Figure S7:** The calibration curves of the prediction model.
**Figure S8:** DCA curves of the prediction model.
**Table S1:** Frequency distribution of metastasis sites by PrBC and PPBC.
**Table S2:** Frequency distribution of known first site of metastasis in TNBC.
**Table S3:** Clinicopathological characteristics of training and testing cohorts.
**Table S4:** Results of multivariate cox regression for training cohort.

## Data Availability

The data that support the findings of this study are available on request from the corresponding author. The data are not publicly available due to privacy or ethical restrictions.
