# Supplementary material for: Clinical and Pathological Characteristics and Prognosis of Breast Cancer During Pregnancy and Postpartum: A Multicenter Retrospective Study
Source: Cancer Rep (Hoboken). 2026 Jun 4;9(6):e70577. doi: 10.1002/cnr2.70577 (PMC13238683; doi:10.1002/cnr2.70577)
Supplement: Supplementary file 1 — Figure S1: Study profile. Figure S2: Analysis of known clinical risk factors associated with distant metastasis in PPBC. Figure S3: Association of childbirth and time since recent childbirth with outcomes. Figure S4: Association of childbirth and time since recent childbirth with outcomes by stage or ER status. Figure S5: Histogram of the coefficients of the selected features. Figure S6: AUCs of the prediction models. Figure S7: The calibration curves of the prediction model. Figure S8: DCA curves of the prediction model. Table S1: Frequency distribution of metastasis sites by PrBC and PPBC. Table S2: Frequency distribution of known first site of metastasis in TNBC. Table S3: Clinicopathological characteristics of training and testing cohorts. Table S4: Results of multivariate cox regression for training cohort. [file CNR2-9-e70577-s001.docx]

**Supplemental material**

Figure S1. Study profile

Figure S2. Analysis of Known Clinical Risk Factors Associated with Distant Metastasis in PPBC.

Figure S3. Association of Childbirth and Time Since Recent Childbirth with Outcomes.

Figure S4. Association of Childbirth and Time Since Recent Childbirth with Outcomes by Stage or ER status.

Figure S5. Histogram of the coefficients of the selected features

Figure S6. AUCs of the prediction models.

Figure S7. The calibration curves of the prediction model.

Figure S8. DCA curves of the prediction model.

Table S1. Frequency Distribution of Metastasis Sites by PrBC and PPBC.

Table S2. Frequency Distribution of Known First Site of Metastasis in TNBC.

Table S3. Clinicopathological characteristics of training and testing cohorts.

Table S4. Results of Multivariate Cox regression for Training Cohort.


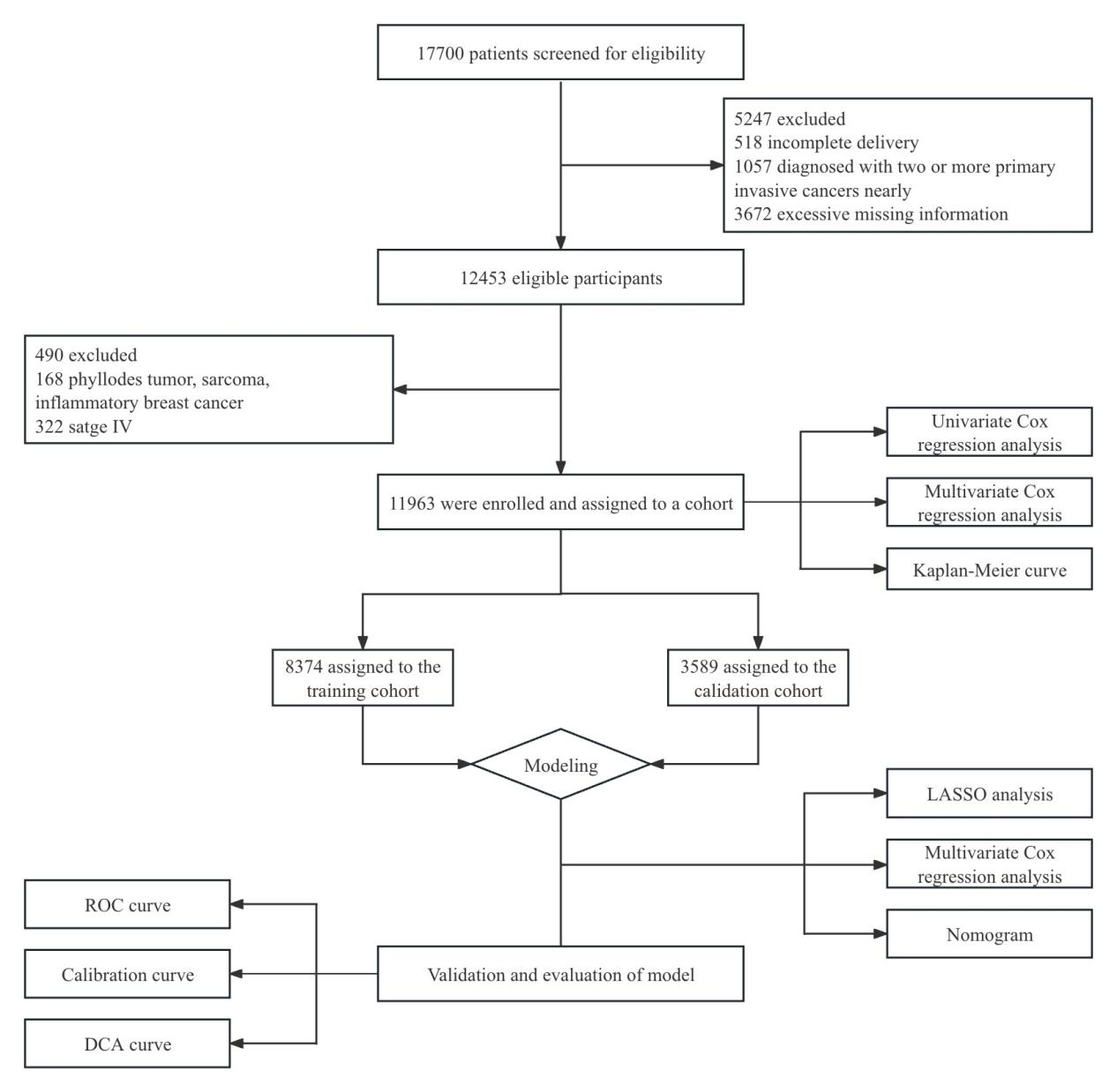


**Figure S1. Study profile.**

*incomplete delivery: missing date of most recent childbirth or unavailable deliver records;

Patients were excluded if key exposure or outcome-related variables were unavailable, including time from most recent childbirth to diagnosis, stage information, survival status, or follow-up time. Because these variables were essential for the primary analyses, complete-case analysis was used, and multiple imputation was not performed.

**Figure S2. Analysis of Known Clinical Risk Factors Associated with Distant Metastasis in PPBC.**

1. Tumor size in all patients. B. percentage of patients with positive lymph nodes at diagnosis. C. Ki67 expression. D. Tumor size in patients with stage I to III.

The error bar is the median of the interquartile range. Significance: * represents *P*<0.05, * * represents *P*<0.01, * * * represents *P*<0.001, ****Indicates *P*<0.0001.

**Figure S3. Association of Childbirth and Time Since Recent Childbirth with Outcomes.**

A, B. All patients DFS and BCSS. C, D. PrBC and PPBC patients DFS and BCSS.

**Figure S4. Association of Childbirth and Time Since Recent Childbirth with Outcomes by Stage or ER status.**

A and C and E. DFS in women with all stage, ER negative diseases or stage III cancer. B and D and F. BCSS in women with all stage, ER negative diseases or stage III cancer.


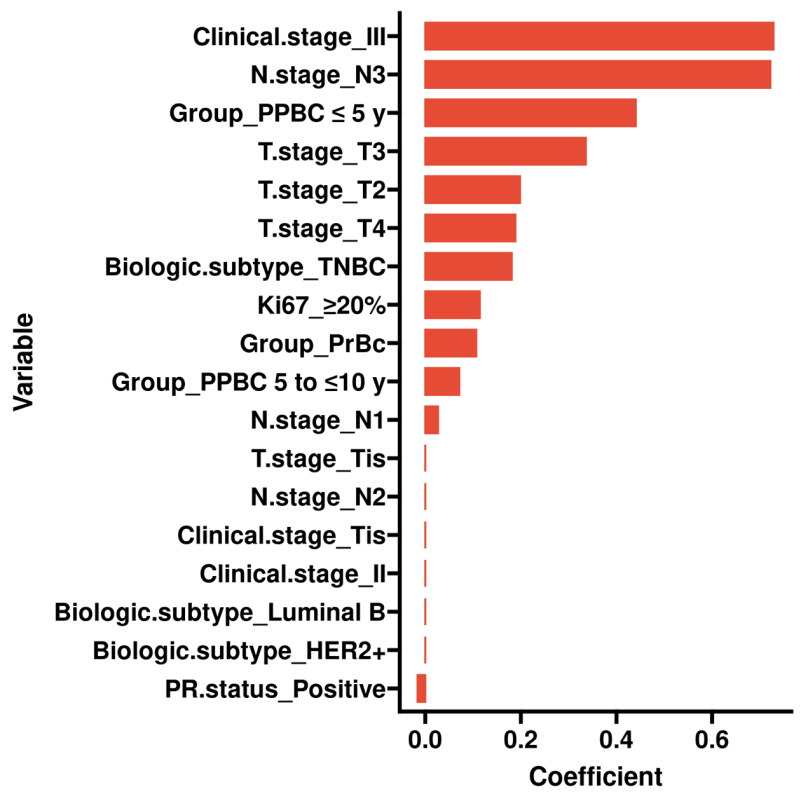


**Figure S5. Histogram of the coefficients of the selected features.**

**Figure S6. AUCs of the prediction models.**

A. The training cohort. B. The test cohort.

**Figure S7. The calibration curves of the prediction model.**

A-C. The training group. D-F. The test group.

**Figure S8. DCA curves of the prediction model.**

A-C. The training group. D-F. The test group.

**Table S1. Frequency Distribution of Metastasis Sites by PrBC and PPBC.**

| **Metastasis Site** | **Total sites**  **(%)** | **PrBC** | **PPBC ≤5** | **PPBC 6 to ≤10** | **PPBC >10** | **Total n of Metastasis (%)** | **PrBC** | **PPBC ≤5** | **PPBC 6 to ≤10** | **PPBC >10** |
| --- | --- | --- | --- | --- | --- | --- | --- | --- | --- | --- |
| Bone | 614 (35.9) | 17 (36.9) | 74 (33.7) | 125 (38.2) | 398 (35.5) | 269 (34.2) | 6 (33.3) | 25 (21.6) | 44 (33.3) | 194 (37.3) |
| Lung | 446 (26.1) | 8 (17.4) | 56 (25.6) | 85 (25.9) | 297 (26.5) | 230 (29.3) | 2 (11.1) | 34 (29.3) | 34 (25.8) | 160 (30.8) |
| Liver | 384 (22.4) | 16 (34.7) | 45 (20.5) | 67 (20.5) | 256 (22.9) | 213 (27.1) | 8 (44.4) | 38 (32.8) | 39 (29.5) | 128 (24.6) |
| Brain and nervous system | 194 (11.3) | 4 (8.7) | 30 (13.7) | 33 (10.1) | 127 (11.3) | 74 (9.4) | 2 (11.1) | 19 (16.4) | 15 (11.4) | 38 (7.3) |
| Kidney | 29 (1.7) |  | 5 (2.3) | 5 (1.5) | 19 (1.7) |  |  |  |  |  |
| Ovary | 16 (0.9) |  | 5 (2.3) | 4 (1.2) | 7 (0.6) |  |  |  |  |  |
| Uterus | 1 (0.1) |  |  |  | 1 (0.1) |  |  |  |  |  |
| Vagina | 2 (0.1) |  |  | 2 (0.6) |  |  |  |  |  |  |
| Pancreas | 5 (0.3) |  |  | 2 (0.6) | 3 (0.3) |  |  |  |  |  |
| Spleen | 6 (0.3) | 1 (2.2) | 2 (0.9) | 1 (0.3) | 2 (0.2) |  |  |  |  |  |
| Stomach | 4 (0.2) |  | 2 (0.9) | 2 (0.6) |  |  |  |  |  |  |
| Appendix | 1 (0.1) |  |  |  | 1 (0.1) |  |  |  |  |  |
| Rectum | 4 (0.2) |  |  |  | 4 (0.4) |  |  |  |  |  |
| Thyroid | 2 (0.1) |  |  |  | 2 (0.2) |  |  |  |  |  |
| Heart | 4 (0.2) |  |  |  | 4 (0.4) |  |  |  |  |  |
| Total | 1712 | 46 | 219 | 327 | 1120 | 786 | 18 | 116 | 132 | 520 |

**Table S2. Frequency Distribution of Known First Site of Metastasis in TNBC.**

|  | **TNBC（n = 104）** | | | | | **non-TNBC（n = 682）** | | | | |
| --- | --- | --- | --- | --- | --- | --- | --- | --- | --- | --- |
| **Known First Site of Metastasis** | **Total n of Metastasis (%)** | **PrBC** | **PPBC ≤5** | **PPBC 6 to ≤10** | **PPBC >10** | **Total n of Metastasis (%)** | **PrBC** | **PPBC ≤5** | **PPBC 6 to ≤10** | **PPBC >10** |
| Bone | 27 (26.0) | 0 (0.0) | 6 (33.3) | 3 (16.7) | 18 (28.1) | 260 (38.1) | 5 (35.7) | 18 (27.7) | 41 (36.0) | 196 (40.1) |
| Lung | 38 (36.5) | 1 (15.0) | 3 (16.7) | 10 (55.6) | 24 (37.5) | 185 (27.1) | 1 (7.1) | 28 (43.1) | 24 (21.1) | 132 (27.0) |
| Liver | 24 (23.1) | 3 (75.0) | 3 (16.7) | 4 (22.2) | 14 (21.9) | 179 (26.2) | 7 (50.0) | 35 (53.8) | 35 (30.7) | 102 (20.1) |
| Brain and Nervous System | 15 (14.4) | 0 (0.0) | 6 (33.3) | 1 (5.6) | 8 (12.5) | 91 (13.3) | 1 (7.1) | 17 (26.2) | 14 (12.3) | 59 (12.1) |
| Total | 104 (13.2)^a^ | 4 | 18 | 18 | 64 | 682 (86.8)^a^ | 14 | 65 | 114 | 489 |

1. Percentages based on total number of with known first metastasis site participants (n = 786).

**Table S3. Clinicopathological characteristics of training and testing cohorts.**

| **Characteristic** | **Training Cohort**  **(N = 8374）** | **Internal Test Cohort**  **(N = 3589）** | **P value** |
| --- | --- | --- | --- |
| Group |  |  | 0.496 |
| PPBC ˃ 10 y | 6,385 (76.2%) | 2,778 (77.4%) |  |
| PPBC ≤ 5 y | 670 (8.0%) | 270 (7.5%) |  |
| PPBC 5 to ≤10 y | 1,245 (14.9%) | 515 (14.3%) |  |
| PrBc | 74 (0.9%) | 26 (0.7%) |  |
| BC family history |  |  | 0.789 |
| No | 7,966 (95.1%) | 3,410 (95.0%) |  |
| Yes | 408 (4.9%) | 179 (5.0%) |  |
| Parity |  |  | 0.366 |
| 1 | 5,497 (65.6%) | 2,403 (67.0%) |  |
| 2 | 2,129 (25.4%) | 883 (24.6%) |  |
| 3 or more | 748 (8.9%) | 303 (8.4%) |  |
| T stage |  |  | 0.712 |
| T1 | 4,037 (48.2%) | 1,760 (49.0%) |  |
| T2 | 3,146 (37.6%) | 1,311 (36.5%) |  |
| T3 | 314 (3.7%) | 128 (3.6%) |  |
| T4 | 101 (1.2%) | 50 (1.4%) |  |
| Tis | 776 (9.3%) | 340 (9.5%) |  |
| N stage |  |  | 0.967 |
| N0 | 5,189 (62.0%) | 2,232 (62.2%) |  |
| N1 | 2,016 (24.1%) | 864 (24.1%) |  |
| N2 | 728 (8.7%) | 302 (8.4%) |  |
| N3 | 441 (5.3%) | 191 (5.3%) |  |
| Clinical stage |  |  | 0.751 |
| I | 2,890 (34.5%) | 1,242 (34.6%) |  |
| II | 3,481 (41.6%) | 1,493 (41.6%) |  |
| III | 1,286 (15.4%) | 530 (14.8%) |  |
| Tis | 717 (8.6%) | 324 (9.0%) |  |
| PR status |  |  | 0.357 |
| Negative | 2,438 (29.1%) | 1,015 (28.3%) |  |
| Positive | 5,936 (70.9%) | 2,574 (71.7%) |  |
| HER2 status |  |  | 0.777 |
| Negative | 5,685 (67.9%) | 2,446 (68.2%) |  |
| Positive | 2,689 (32.1%) | 1,143 (31.8%) |  |
| Ki67 |  |  | 0.763 |
| ˂20% | 2,798 (33.4%) | 1,189 (33.1%) |  |
| ≥20% | 5,576 (66.6%) | 2,400 (66.9%) |  |
| ER |  |  | 0.979 |
| Negative | 1,565 (18.7%) | 670 (18.7%) |  |
| Positive | 6,809 (81.3%) | 2,919 (81.3%) |  |
| Diagnosis age |  |  | 0.730 |
| 40-55 | 6,160 (73.6%) | 2,651 (73.9%) |  |
| ≤40 | 2,214 (26.4%) | 938 (26.1%) |  |
| Age at menarche |  |  | 0.924 |
| ˂ 13 | 1,203 (14.4%) | 518 (14.4%) |  |
| ≥ 13 | 7,171 (85.6%) | 3,071 (85.6%) |  |
| Histologic subtype |  |  | 0.105 |
| Ductal | 7,797 (93.1%) | 3,342 (93.1%) |  |
| Lobular | 249 (3.0%) | 87 (2.4%) |  |
| Other | 328 (3.9%) | 160 (4.5%) |  |
| Biologic subtype |  |  | 0.884 |
| Luminal A | 1,849 (22.1%) | 788 (22.0%) |  |
| HER2+ | 719 (8.6%) | 318 (8.9%) |  |
| Luminal B | 4,961 (59.2%) | 2,135 (59.5%) |  |
| TNBC | 845 (10.1%) | 348 (9.7%) |  |

**Table S4. Results of Multivariate Cox regression for Training Cohort.**

| **Characteristic** | **N** | **Event N** | **HR** | **95% CI** | ***P* value** |
| --- | --- | --- | --- | --- | --- |
| Group |  |  |  |  |  |
| PPBC ˃ 10 y | 6,385 | 594 | — | — |  |
| PPBC ≤ 5 y | 670 | 121 | 2.13 | 1.75, 2.59 | <0.001 |
| PPBC 5 to ≤10 y | 1,245 | 158 | 1.50 | 1.26, 1.79 | <0.001 |
| PrBc | 74 | 17 | 2.17 | 1.33, 3.55 | 0.002 |
| T stage |  |  |  |  |  |
| T1 | 4,037 | 305 | — | — |  |
| T2 | 3,146 | 428 | 1.55 | 1.27, 1.89 | <0.001 |
| T3 | 314 | 78 | 2.06 | 1.53, 2.79 | <0.001 |
| T4 | 101 | 31 | 2.44 | 1.58, 3.77 | <0.001 |
| Tis | 776 | 48 | 0.77 | 0.28, 2.12 | 0.618 |
| N stage |  |  |  |  |  |
| N0 | 5,189 | 364 | — | — |  |
| N1 | 2,016 | 228 | 1.54 | 1.24, 1.91 | <0.001 |
| N2 | 728 | 147 | 1.92 | 1.25, 2.97 | 0.003 |
| N3 | 441 | 151 | 3.89 | 2.53, 6.00 | <0.001 |
| Clinical stage |  |  |  |  |  |
| I | 2,890 | 167 | — | — |  |
| II | 3,481 | 361 | 0.96 | 0.72, 1.28 | 0.786 |
| III | 1,286 | 316 | 1.31 | 0.80, 2.16 | 0.282 |
| Tis | 717 | 46 | 1.50 | 0.53, 4.24 | 0.442 |
| PR status |  |  |  |  |  |
| Negative | 2,438 | 299 | — | — |  |
| Positive | 5,936 | 591 | 0.84 | 0.70, 1.01 | 0.061 |
| Ki67 |  |  |  |  |  |
| ˂20% | 2,798 | 221 | — | — |  |
| ≥20% | 5,576 | 669 | 1.16 | 0.92, 1.47 | 0.203 |
| Biologic subtype |  |  |  |  |  |
| Luminal A | 1,849 | 138 | — | — |  |
| HER2+ | 719 | 78 | 1.08 | 0.73, 1.60 | 0.704 |
| Luminal B | 4,961 | 538 | 1.23 | 0.92, 1.63 | 0.160 |
| TNBC | 845 | 136 | 1.66 | 1.17, 2.37 | 0.005 |
